# M. Andral on Hyperemia

**Published:** 1832-01-01

**Authors:** 


### On Hyperæmia. By *M. Andral.*

M. Andral's work contains a mine of information, research, and deep thought, which the translation of Drs. Townsend and West have laid open to the mere English reader—provided they take the trouble to explore it.

But we are sorry to say that light reading and medical politics occupy so many of the leisure hours of medical men in this country, that only a very small proportion of them "drink deep" at the fountain-head of knowledge. If an easy or a quackish mode of treating any important disease, as, for example, fever, be proclaimed, every one hastens to devour the discovery, and take a note of the remedy. But if a deep and laborious investigation into the nature and causes of the malady be advertised, very few indeed will bestow the time or the expense necessary for its perusal. Too many appear to act under the impression, that every study, except of *curing* a complaint, is useless labour; forgetting, or rather not knowing, that we cannot learn the cure, without first learning to discriminate the disease. Almost every malady has its stages and varieties, demanding nearly opposite modes of treatment; though the quack and the routinist hold out but one process for all! Notwithstanding these discouraging circumstances, we shall lay a full analysis of the most interesting articles in M. Andral's work before our readers, hoping that a great many of them will be induced to peruse both the abstracts and the original.

The portion which we select for this article is HYPERÆMIA, occupying the first chapter in "lesions of the circulation." M. A. defines this state to be, "a preternatural accumulation of blood in the capillary vessels." This general condition he subdivides into the four following species, viz. 1, Active or *sthenic* hyperæmia, produced by irritation:—2, Passive or *asthenic* hyperæmia, resulting from diminished tone in the capillary system:—3, *Mechanical,* from obstacle to the venous circulation:—4, *Cadaveric,* or *post mortem.*

### I. ACTIVE, OR STHENIC HYPERÆMIA.

Some local congestions, our author observes, are compatible with a healthy state of the system; as exemplified by blushing under mental emotion, and redness of the surface under corporeal exertion. But some others approach nearly to a state of disease; when, for example, the skin becomes red and congested from exposure to too high a temperature, or to irritating substances. Slight congestions thus induced, do not interfere with the functions of the parts or of the general system; but if the excitation be great, or long continued, a true pathological condition is gradually formed, attended with pain and functional derangement, calling various morbid sympathies into action. No line of demarcation can be drawn between these affections. They slide into one another by imperceptible steps and gradations.

"The formation of active congestions by no means infers an excessive proportion of the general mass of blood circulating in the system. On the contrary, morbid anatomy has clearly proved that active hyperæmia occurs as frequently in debilitated individuals, whose blood is neither abundant in quantity, nor rich in quality, as it does in persons of the most plethoric temperament; the only apparent difference consisting in the local and general symptoms attendant on those congestions. The same remark is applicable to the influence of age; with this difference, however, that, at different periods of life, the seat of the hyperæmia varies, as well as the symptoms by which it is attended, but at all ages, the frequency of the formation of active congestions is nearly equal.

When a hyperæmia is formed in any one organ, it has a remarkable tendency to extend and establish itself in other parts of the system; for they are all closely and intimately connected. The capillary system, when deranged in any one point, is liable to become generally disordered, in which case it presents one of two phenomena: either the original congestion is propagated to other parts, or, at the same time that one or more organs are in a state of hyperæmia, some other parts of the body (by virtue of a species of compensation established between the circulating forces of the capillary system) receive less blood than natural, and fall into a state of temporary, or even permanent anæmia. Thus when the mucous membrane of the stomach is affected with hyperæmia, the cutaneous surface is sometimes minutely injected, while in other cases it is discoloured, and in others again, pale as death: the brain and its investing membranes may likewise, in one case, present unequivocal marks of violent congestion, and in another be found almost exsangueous, and decidedly paler than natural.

These pathological observations afford satisfactory explanations of several morbid phenomena. Thus, for instance, they enable us to understand how the delirium, convulsions, and other nervous disorders, so frequently supervening during attacks of acute gastro-enteritis, are in some cases produced by the repetition in the cerebro-spinal system, of the congestion originally formed in the mucous membrane of the intestinal canal; whilst in others, the same symptoms depend on the exsangueous state of the nervous system, resulting from the circumstance of the blood's accumulation in the organ originally congested.

When a hyperæmia is formed in any part of the system, if any one organ be at the time in a state of disease, or have previously been so, it is that organ which the hyperæmia has the greatest disposition to affect secondarily; hence it is, that when, from any cause whatever, a local congestion is formed in any part of the body, we observe as secondary phenomena, palpitations, dyspnœa, hæmoptysis, gastric symptoms, hæmaturia, or menorrhagia; according as the heart, lungs, stomach, kidneys, or uterus, are, or have been, diseased, and thus rendered more susceptible of secondary hyperæmia." 19.

When previous disease has not predisposed some particular organ to secondary hyperæmia, there is generally a determinate order in the disposition of organs to be thus secondarily affected. The brain and spinal cord appear to be most disposed to sympathetic congestions—then the alimentary canal —the lungs—the heart—and lastly the skin. Much modification and variety, however, will be found, according to individual constitution, &c.

When the primary hyperæmia is rapidly developed, those secondarily formed are generally acute also; and from the rapidity of their formation, or the number of parts affected simultaneously, they often produce as urgent and threatening symptoms as could result from much more serious organic diseases.

"Thus, the violent dyspnœa, almost threatening instant suffocation, which not unfrequently supervenes in an acute attack of gastro-enteritis, often leaves no other trace of organic derangement, than a slight sanguineous congestion of the pulmonary tissue, insufficient to prevent the access of air; in like manner, notwithstanding the host of nervous symptoms, which complicate the progress of almost every attack of acute inflammation, the body frequently presents, on examination after death, no morbid appearance except a slight congestion of the vessels of the brain, so slight indeed, that it may in some cases fairly be questioned, whether this congestion be really the cause of the great derangement of the cerebral functions, or whether it be not rather an effect resulting from the deranged action of the nervous system, especially as all those nervous symptoms are sometimes observed when no such congestion can be discovered." 21.

The secondary hyperæmias may, from their commencement, assume the chronic character of the original hyperæmia, and, whilst their progress is slow, and their symptoms insidious, their effects may be equally fatal.

"Sometimes, after having long continued stationary in their chronic form, they suddenly burst forth with all the violence of an acute and newly-formed disease; in other cases, during the progress of the chronic affection, a new and acute hyperæmia seizes on an organ which till then had remained free from disease.

This supervention of acute hyperæmia is one of the most frequent causes of the sudden deaths which occur during the progress of various chronic affections; the lungs and alimentary canal are the parts most liable, in chronic affections, to both these species of hyperæmia, namely, aggravation of the chronic, and the supervention of the acute form." 22.

When the secondary hyperæmia is formed, the primary one is either unaffected—exasperated—or mitigated. The last is the least common.

"When a patient has lost a large quantity of blood in a short space of time; when, during convalescence from a tedious illness, he has been long kept on low diet; or when, after an attack of acute inflammation, he continues to labour under the disease in a chronic form; whenever, in short, the system has been much exhausted without adequate means being taken to recruit its losses, it frequently happens that the sensibility of the nervous system to impressions is increased, in the same proportion as the muscular strength and quantity of blood are diminished. Under such circumstances, a hyperæmia attended with the least degree of pain, may excite the most alarming derangement in the functions of the nervous system. I have seen, in a case of this kind, the bite of a single leech produce symptoms of tetanus; it is scarcely necessary to add, that the application of more powerful irritants, such as cupping-glasses, blisters, or sinapisms, is still more decidedly contraindicated in such cases. To this MORBID SENSIBILITY of the nervous system must be attributed the injurious effects so frequently observed to follow the application of revulsives to persons debilitated by copious venesection or protracted abstinence from nutritious diet; not that the hyperæmia attended with more or less pain, that is produced by the revulsives, directly aggravates the original hyperæmia, but that it produces a violent effect upon the nervous system, which in its turn, reacts upon the primary disease, and thus aggravates those symptoms which it was intended to relieve.

This exquisite sensibility of the nervous system is not exclusively confined to persons labouring under chronic disease, or reduced by tedious convalescence; there are some individuals in whom this state of the nervous system is constitutional: they are generally persons of a delicate frame, and whose muscular system is imperfectly developed. In such cases, all our attempts to remove local congestions by abstraction of blood, only give an increased predominance to the nervous symptoms, the repetition of the blood-letting serving but to increase the convulsions, coma, delirium, &c." 24.

If the above be true, the local congestion should not engross the whole of our attention; and hence the importance of studying the various modifications dependent on constitutional idiosyncrasy, as far as the nervous system is concerned. This system is liable to be primarily affected; and when deranged in its functions and in the influence which it exerts over the other systems, it may and does, in its turn, produce in them either temporary or permanent congestions, thus laying the foundation of every species of organic disease. In this way, an affection at first purely nervous, may be subsequently transformed into a hyperæmia, and finally produce an extensive alteration in the organization of the part—changes, however, which are not always or uniformly characterized by a corresponding alteration in the symptoms.

When several hyperæmias are formed simultaneously in different organs, they are sometimes produced by one another—but sometimes they are quite independent, though owing to one and the same cause.

"Thus, in measles and scarlatina, two congestions uniformly exist together; one, on the cutaneous surface, the other, in certain portions of the mucous membrane. It would be absurd to say that, in these diseases, the cutaneous produced the mucous hyperæmia, or *vice versa:* they are both necessary effects of one and the same cause; the manifestation, as it were, of the morbid state of the system produced by the introduction of the contagious poison.

This co-existence of several hyperæmias appears to be one of the most constant effects of the introduction of any deleterious principle into the circulation. It is constantly observed in all contagious and infectious diseases, termed typhoid, or pestilential; and likewise in animals that have been made to swallow poisons susceptible of absorption, or when putrid substances have been injected into their veins. The poison, when conveyed into the circulation and mixed with the blood, produces three grand effects, which may either exist singly or in combination. 1. It alters the blood itself, and renders it to a greater or less extent unfitted to support the nutrition and life of the different organs. 2. It modifies the functions of the nervous centres. 3. It produces irritation, alteration of nutrition, and hyperæmia, of the organs to which it is distributed in its vehicle, the blood. This last effect is however the least constant of the three, and great functional derangement may be produced independently of it. Hence it follows, that if we would form a correct and precise idea of the nature and treatment of those diseases which are produced by miasmata or other poisons, we must consider the hyperæmia formed in the mucous membrane of the intestines, or elsewhere, as simply one of the elementary ingredients of these diseases; an ingredient which may be absent and yet the disease be neither less rapid in its progress, nor fatal in its termination on that account." 27.

Sometimes a hyperæmia exists for a considerable period in an organ, without producing any alteration in its nutrition or secretions, except perhaps some increase or diminution in their quantity. In other cases, the organs which have been the seat of hyperæmia, undergo various alterations in their nutrition and secretion.

"Congestions of different degrees of intensity and duration occasionally assume an intermittent type, and recur, at longer or shorter intervals, in the immediate vicinity or in the substance of a tissue which has already undergone some chronic alteration in its structure and organization. The recurrence of those attacks of hyperæmia often renders evident the existence of organic lesions, which, during the absence of the sanguineous congestion, were either wholly latent, or only revealed by the most obscure and equivocal symptoms; every return of the hyperæmia in such cases invariably aggravates the organic disease, and accelerates its progress. The knowledge of this fact explains the utility of blood-letting in such circumstances; the effects of the newly-formed hyperæmia are thereby diminished, and although the original organic disease be not removed, or even diminished, it is relieved from the acute symptoms induced by the supervention of the hyperæmia, and is thus brought back to its original stationary condition." 31.

We must not, however, suppose that local congestions can be uniformly removed by blood-letting. A hyperæmia may exist singly and independent of any organic alteration; and yet refuse to yield to local or general depletion. By these depletions the congested organ is relieved of a part of its superabundant fluid; but by such means we cannot remove the unknown cause by which the hyperæmia was originally developed. If this exciting cause of congestion—this thorn of Van Helmont—be powerful in its action, we may drain away the vital fluid, yet the last drop of blood in the body will, in despite of all our bleedings, obey the summons of the irritating cause, and rush to the part affected.

"The modern Italian school has fully recognized the truth of this principle. Convinced of the inefficacy of blood-letting for the removal of the primary cause of congestions, it has endeavoured to discover remedies capable of directly combating this cause. How far the *contra-stimulant* medicines really fulfil this indication, it is neither consistent with the object of this treatise, nor important for our present purpose, to determine; my only object being to establish the primary indication, which should present itself to our consideration whenever the hyperæmia does not depend on a simple irritating cause, externally applied. This indication, I repeat it, consists in combating the cause by which the congestion was produced. Experience alone will enable us to decide whether this indication can be accomplished. We may however remark, as connected with this subject, that observation has already led us to discover, in cinchona, a remedy eminently adapted to prevent the recurrence of intermitting hyperæmias; and that the writings of Tommassini, and his followers, contain many strong facts in favour of the contra-stimulant doctrine; facts which we are by no means entitled to reject, because we cannot reconcile them with our preconceived ideas, and which must eventually be decided by the test of experience, and not by their conformity with any particular theory. These observations may serve to prove that all our therapeutical indications cannot be derived from morbid anatomy, and that in many important questions connected with this subject, no information whatever is to be derived from that source." 33.

The phenomena of hyperæmia, our author thinks, both in the healthy and morbid states, prove incontestibly that the blood in the capillary vessels is withdrawn from under the influence of the heart, and that its movements and local determinations are regulated by forces inherent in the capillaries themselves. The nervous system modifies the action of these forces, as is observed in the act of blushing. Our author next adverts to various experiments on the state of the circulation in inflammation. In the first degree of hyperæmia, he contends that the vessels are in a state of contraction, and the circulation accelerated. In the second degree, which succeeds to the first, the vessels dilate—the blood circulates more slowly—its particles tend to coalesce—and the whole mass seems disposed to coagulate. From the condensation and unusual accumulation of the blood, the part affected assumes a deep red colour, which subsequently changes to a brown shade, as the circulation of the part becomes more completely suspended. In the third and last degree of hyperæmia, the blood becomes perfectly stagnant, and the seat of the hyperæmia assumes a yet deeper shade of brown, and finally becomes quite black.

"Let us now endeavour to investigate the nature of those forces under the influence of which the capillary vessels, that were at first contracted, become subsequently dilated. Does the force by which the blood is impelled from all parts towards the seat of irritation, contrary to its natural course and the laws of gravity, reside in the blood itself? Is the dilatation of the vessels a passive result of the mechanical distention which they undergo from the unusual afflux and accumulation of blood? or does it rather proceed from the diminished elasticity of their parietes, resulting from some alteration in their texture? or, lastly, does it arise from an expansive force residing in the coats of the vessels; a force analogous to that which appears to exist in the parietes of the heart (whose dilatation is decidedly not a passive phenomenon) and in the erectile tissues?

The analogy is perhaps stronger than might at first be expected, between the phenomena which occur in an organ affected with active hyperæmia, and those which take place in a tissue in a state of erection. One material difference may however be noticed, namely, that in the erectile tissue the parts are naturally so constructed, and disposed, as to admit of a sudden accumulation of blood under the influence of certain physiological conditions; whereas the natural texture of an organ not erectile must first undergo some modification, before it is capable of receiving and retaining more blood than it is ordinarily supplied with; hence arise various derangements of function in the organ congested, and alterations in its nutrition, secretion, and sympathetic relations with other organs.

As these considerations are not so much matters of fact as of speculation, I shall not urge them further at present, and shall conclude this part of my subject with the observation, that even if all hyperæmias are identical at their commencement, if they all uniformly consist in an unusual afflux of blood towards some point, accompanied by contraction of the vessels, and increased rapidity in the local circulation; if, I say, in this first stage of their existence, they are simply the phenomena of the healthy condition of the part in a state of exaggeration, a second period sooner or later succeeds, in which the phenomena can no longer be considered as such; at this period commences the development of the different alterations of texture, and of the various morbid secretions, which we cannot at all conceive to be produced by the simple augmentation of organic action." 44.

Hyperæmia, instead of being confined to one or more organs, sometimes exists in every organ in the body. The general capillary system is then overloaded with blood, and the whole body is in a state of plethora. The essential character of this state appears to consist in the formation of more blood than is necessary for nutrition and secretion. This superabundance of blood becomes a permanent source of excitation to the solids, while the vital fluid itself has a tendency to accumulate in different organs, forming local congestions of various degrees of intensity.

"Under the influence of this state of general hyperæmia, serous effusions unattended with pain or other symptoms of inflammation, take place into the cellular tissue and into the different cavities lined with serous membranes, especially the abdomen. It appears to me highly probable that these dropsical effusions which are generally denominated *active*, are simply the mechanical result of the over-distention of the vessels, which allow the serous portion of the fluid by which they are over-distended, to transude through the parietes of their capillary ramifications. In confirmation of this view of the subject, I may cite the observation, that if a large quantity of water be injected into the veins of an animal, without having first withdrawn blood from his system, serous effusions are quickly formed; whereas, if the mass of blood be diminished by venesection before the water be injected, that fluid is gradually and almost imperceptibly eliminated. Besides we know from actual experience, that those dropsies usually termed active, which are combined with a state of general hyperæmia of the system, are constantly relieved, and not unfrequently altogether removed, by the use of the lancet." 47.

In these cases of universal hyperæmia, where the whole system is overexcited by the excessive supply of blood, the sympathies which associate the different organs, are rendered more active, and inordinate re-action ensues. The functions of the nervous system are disordered—the temperature of the surface elevated—the different secretions variously modified—the pulse augmented in strength and frequency—and, in short, all the phenomena of fever developed. This fever may be ephemeral, or it may continue for many days. It may run a course, as simple continued fever; or, the intensity of re-action among the different organs may give rise to alarming symptoms, and various nervous phenomena, with prostration of strength, and false adynamia. Or, lastly, some one organ may become more especially affected, and the disease, which was universal in its commencement, is converted into a local affection.

"The morbid state which I have now described, and to which may be referred some of the species of continued fever described by the older nosologists, may terminate in recovery or death. When the termination is favourable, the symptoms gradually improve, as the superabundant quantity of blood, the original source of all the accidents, is diminished by abstinence and blood-letting. When death ensues, the *post-mortem* examination generally exhibits traces of a well-marked inflammation of one or more organs; this inflammation seems to have taken place subsequently to the commencement of the febrile paroxysm, at least the symptoms would lead us to this conclusion. But on other occasions no trace of inflammation can be discovered, and the only morbid appearance consists in a simple accumulation of blood in the capillaries of different organs, their texture remaining perfectly unaltered. These slight congestions, affecting simultaneously several organs, may, by the various morbid sympathies which they excite, produce as violent and formidable symptoms as the most serious organic lesion of any individual organ. In such cases where are we to assign the origin of the disease? Wherever the blood is distributed, there derangement of function is found. In the blood, then, indisputably resides the first cause of the disease; the lesion of the solids is only a secondary affection, but may notwithstanding become, during the progress of the disease, the predominant affection, and give rise to many and formidable accidents." 49.

### II. ASTHENIC HYPERÆMIA.

The violet colour which we frequently see on the surface of the legs and feet of elderly people is probably owing to diminished energy of the capillary circulation. It appears that in these cases the blood, having arrived at the termination of the arterial branches, has a tendency to stagnate wherever the force of the heart and arteries is diminished. This is proved by the fact that the horizontal position will remove these discolorations.

"In some of these cases, the blood which passes from the arteries into the capillaries of the feet, returns but in small quantities by the veins, and gradually accumulates to such an extent, as to oppose an effectual obstacle to the arrival of a fresh supply of arterial blood from the heart. The blood thus accumulated in the capillaries, being arrested in its course, coagulates, and obliterates the cavities of the vessels, so that their calibre is filled with coagulated blood, which is often found advancing towards organization. The same series of phenomena must then ensue, as when the blood was accumulated in the capillary vessels under the influence of an acute hyperæmia, (see the preceding article;) namely, the blood becomes black, is no longer adapted to maintain the life of the part, and gangrene supervenes.

Such is the true pathology of the disease termed *gangrena senilis*. There is at first, in the most dependent portion of the limb, passive stagnation of blood in the capillaries, and, in consequence of the mechanical obstacle to the circulation thus formed, coagulation of the blood which arrives by the arteries, and, as a necessary consequence of these two phenomena, gangrene of the feet and legs."* 51.

The existence of a true asthenic hyperæmia appears to our author to be fully established in the case just described—and ought to be borne in mind by those who conclude that, because a part is more red than natural, it must necessarily be affected with *active* hyperæmia. By covering the part with emollient poultices we only aggravate the disease; whereas by applying active stimulants we rouse the languid circulation of the capillaries. Before proceeding to consider whether the redness observable in the internal organs may not, in like manner, occasionally depend upon passive congestion, it may be worth while to examine whether other instances might not be brought forward to illustrate the existence of asthenic hyperæmia in the surface of the body; especially as some modern pathologists have thought proper to deny its existence altogether.

"During the course of certain acute diseases, in which the functions of the nervous system are more or less seriously deranged, the application of the slightest irritation to the cutaneous surface is sufficient to convert the red colour, which the parts previously presented, into a violet, brown, or even black colour; and thus substitute gangrene in the place of a simple sanguineous congestion. No doubt in such cases the gangrene was preceded by active hyperæmia; but does it necessarily follow from thence that the gangrene was produced by excess of irritation? To me it appears much more probable, that, in consequence of some peculiar modifications of the nervous influence, the blood, after accumulating for some time in a part of the cutaneous surface, at length ceased to move, and became perfectly stagnant, because the capillary vessels being deprived of the nervous influence which should regulate their functions, could no longer expel their contents; and in this way a passive hyperæmia was formed where an active hyperæmia had previously existed. The frequency of gangrene in these cases is directly proportional to the alteration of the nervous influence. Its occurrence is most common in the various epidemics of the plague and typhus fever, in which its appearance is not necessarily preceded by increased vascular action, or active hyperæmia; for it constantly happens that several spots in the cutaneous surface turn spontaneously red, then brown, and finally form a gangrenous eschar." 53.

This explanation of gangrene simplifies, says M. Andral, therapeutics, and confirms the propriety of the old practice of covering with powdered cinchona, &c. the red patches, excoriations, and sores which occur during the progress of fevers, whenever their surfaces presented a grey or brownish colour.

"Asthenic hyperæmia also occurs in the external mucous membranes, either as a primitive affection, or consecutively to an attack of active hyperæmia. Thus after the mucous membrane of the eye has been for some time affected with an active congestion of greater or less intensity, three cases may present themselves: 1. The redness of the conjunctiva may disappear altogether. 2. It may continue indefinitely, though in a less violent degree; but the ill effects which are constantly produced by the application of stimulants, prove that the congestion

---

* "M. Cruveilhier succeeded in producing gangrene of a limb, by injecting the minute arterial ramifications of the part with quicksilver."

is still kept up, under the influence of some irritation. 3. There are other cases in which the capillaries of the conjunctiva continue minutely injected, and appear dilated and varicose ; but their colour is a deeper shade of brown, is rather increased than diminished by emollient applications, and often disappears altogether under the use of stimulants. How then is the action of these irritating agents to be explained ? Simply thus ; they stimulate the relaxed debilitated vessels of the conjunctiva, restore their natural tone and elasticity, enable them to propel the blood as quickly as they receive it, and thus dissipate the appearance of increased vascularity.

In this last case then, the hyperæmia was asthenic, while in the other two, it was sthenic ; thus we see that, when we propose treating a congestion on the stimulant plan, the question to determine is, not whether the hyperæmia is acute or chronic, but whether it is sthenic or asthenic. It matters little whether the blood flows towards the part congested for a single day or for a series of months ; if irritation be the cause, the application of any description of stimulus will be injurious ; if, on the contrary, the congestion be kept up solely by the passive dilatation of the vessels, stimulants will then act beneficially, by restoring to the over-distended vessels the power of re-acting on their contents." 56.

The mucous membrane lining the mouth presents, in scorbutic individuals, another example of asthenic hyperæmia; and as it is assumed as an axiom that asthenic hyperæmias may be formed in external parts, the presumption is strong that they may also exist in internal organs. The author commences this important investigation with the lungs. It is acknowledged that no organ is more frequently affected with active hyperæmia than the lungs ; but still M. Andral insists that they are susceptible of other forms of congestion. Thus, he observes, no person will refuse to admit as an instance of asthenic hyperæmia, those sanguineous congestions of the pulmonary parenchyma which so constantly occur in the agonies of death. It is evident in these cases, that the blood driven into the capillary vessels of the pulmonary artery and veins no longer receives from these vessels the impulse that should propel them onwards to the left side of the heart. The lungs are therefore gorged, just as in those animals whose eighth pair of nerves has been divided, or in persons labouring under an apoplectic fit.

" In all these cases the activity of the capillary circulation is diminished, in consequence of the diminished energy of the nervous influence ; and yet the morbid appearances found on dissection are precisely the same as are presented after inflammation ; namely, a great accumulation of blood in the vessels, and in the small bronchial tubes a quantity of serous fluid mechanically filtered from the blood. We should learn from this example not to place too implicit reliance on the anatomical characters of a morbid lesion, as a criterion for deciding the nature of the disease.

But this passive congestion of the lung is not confined to the last moments of the mortal agony. Can we not, for instance, detect its existence in certain cases of convalescence from acute pneumonia, in which a slight degree of dyspnœa remains, and a '*râle crepitant*' continues audible, although the thorax has resumed its natural sound on percussion ? No doubt these symptoms often depend on a trace of the original inflammation still lurking in the part, and not yet subdued ; but I have also seen similar cases, in which the pulmonary congestion continued for a length of time stationary, notwithstanding the employment of antiphlogistics and revulsives, and yet yielded immediately to the use of tonics, such as the decoction of polygala or cinchona." 59.

M. Andral thinks it reasonable to conclude that those substances, when absorbed and carried into the blood, produce the resolution of the pulmonary congestion, either by directly stimulating the coats of the pulmonary vessels, or else by exciting the centres of the nervous system, and restoring their natural influence over the lung and its circulation. And as asthenic succeeds sthenic ophthalmia, why may not the same occur in pneumonia.

"I am also disposed to think that in some other conditions of the œconomy, such as severe scorbutic affections, the lungs may be passively congested as well as the gums and certain portions of the cutaneous surface. This at least I am sure of, that in four cases of severe scurvy, two of whom I attended at the Hôpital des Enfans, a third at La Charité, and the fourth in private, all of whom were affected with constant dyspnœa unattended with other symptoms of pulmonary disease, I found, at the *post-mortem* examinations, the lungs not altered in texture, but gorged with an enormous quantity of blood, remarkable for its extreme tenuity and bright rose colour, and presenting the appearance of water slightly tinged with red. A fluid precisely similar in appearance was effused into the cavities of the different articulations; the spleen and liver were also filled with it: in two of these cases several ecchymoses were found between the tunics of the alimentary canal, and in all of them several patches of the subcutaneous and intermuscular cellular tissue, as well as of the skin itself, were infiltrated with blood.

Have we not, again, instances of asthenic hyperæmia affecting the mucous membrane of the bronchia, in persons labouring under chronic catarrh, whose symptoms are relieved or altogether removed by the use of tonic medicines? On the contrary, in those individuals labouring under the same disease, whose catarrhal symptoms, however chronic, are uniformly aggravated by the use of stimulants, the sthenic character of the original hyperæmia would seem to be still preserved." 61.

If these reasonings be founded in truth, M. Andral thinks we may fairly conclude that many of those red patches found after death in the mucous membrane of the stomach and intestines, are simply the result of passive congestion formed during the life of the individual.

### III. Mechanical Hyperæmia.

This, of course, occurs in cases where impediments are thrown in the current of the circulation, either from simple gravitation—defect of proportion between the respective cavities of the heart—compression or obliteration of a venous trunk—or any obstacle to the passage of the blood through the capillaries.

"When the mechanical hyperæmia is carried to a certain extent, other phenomena may arise as its consequence. Thus, the serous portion of the blood, or even pure blood, may escape from the over-distended vessels, just as water or any other liquid transudes through the permeable sides of a vessel in which it suffers compression. To this source are to be referred several hæmorrhages and dropsies produced by simple transudation in a tissue mechanically congested; and although these effusions have really nothing active in their nature, yet are they considerably diminished, and sometimes altogether removed, by blood-letting, which in such cases acts in a manner purely mechanical, by removing from the vessels the fluid by which their parietes were kept in a state of over-distention. These pathological observations are well exemplified in the majority of those cases of hæmoptysis, hæmatemesis, ascites and other effusions, which are connected with organic disease of the heart." 68.

M. Andral acknowledges that, in the dead body, it is often impossible to distinguish the mechanical hyperæmia from the two other species that have been described—the arborescent appearance of the vessels, and the uniform red colour being common to all the species of hyperæmia. We must, therefore, attend less to the appearances of the part congested, than to other circumstances—such as the kind of death which the individual has died—the nature of the preceding disease—and the state of the other organs.

### IV. HYPERÆMIA FORMED AFTER DEATH.

That sanguineous congestions are really formed after death, on the surface of the body, may be proved to demonstration. The several steps of their formation may be traced, and the causes which favour their production appreciated. And if sanguineous congestions can be thus formed, after death, on the external surface of the body, analogy leads us, *à fortiori*, to conclude that similar congestions may take place in those tissues which are situated in the interior. In the last moments of life, the blood deserts the surface, and concentrates in the internal organs.

"Observation confirms the justice of this reasoning; for if the body of an animal be opened immediately after death, and the state of the organs as regards their colour and the disposition of the blood, be at the time carefully noted, and if those same organs be again examined at successive intervals, the blood will be found to accumulate gradually in different parts, which immediately after death contained no more than the rest; parts naturally white will turn red; vessels will become visible where not a trace of vascularity could be previously observed; there will be developed a coloration of a uniform red tint, disposed in isolated spots, long stripes, or broad patches; the blood will be seen escaping from its vessels, and forming more or less extensive effusions around them, or else soaking through the neighbouring tissues and imparting its colour to them; and, lastly, the colouring matter of the blood will be seen uniting, in the different serous cavities, and in several points of the cellular tissue, with the colourless albuminous fluid which had been previously effused, or else seen transuding through the tunics of the vessels with the serum, if this fluid had not been previously effused separately. At last, when the body, no longer endowed with life, begins to decompose under the influence of those laws which govern all inert matter, various gases are disengaged, which, traversing the coats of the minute vessels, impart an unusual colour to the blood, just as they are found to do in the case of the experiment where they act on this fluid through the coats of the bladder in which it is contained. In this manner are produced the different brown, livid, and greenish hues observable on bodies undergoing putrefaction. The concave surface of the liver is generally the first part to present this appearance, in consequence of its vicinity to the transverse arch of the colon, which usually contains a large quantity of those gases, and also in consequence of the vast proportion of blood contained after death in the capillaries of the liver, which is thus placed in the most favourable circumstances to undergo these chemical alterations: it is not until after the liver and adjacent parts are thus coloured, that the abdominal muscles and subsequently the integuments present the same appearances. Theory might easily have led us to anticipate these results of observation." 74.

In summing up the causes which produce hyperæmia after death, M. Andral is led to recognize several genera and species which he arranges as follows:—

1st GENUS. Hyperæmia produced in articulo mortis, in consequence of the contractility of tissue which resides in the small arteries after the cessation of the heart's action. 2d Genus. Hyperæmia produced at a certain period after death—a genus comprehending three species, viz. hyperæmia by position—by transudation—by chemical affinities. After many interesting remarks on these genera and species, M. Andral goes on to observe, that there is one phenomenon which is liable to succeed to all these species of hyperæmia, namely, the escape of blood from its vessels, and its effusion, either on the free surface of the membranes, into the areolæ of the cellular tissue, or into the parenchymatous texture of the different organs, where it causes, by its presence, a kind of disorganization. The blood likewise escapes from its vessels, in those hyperæmiæ which are formed after death, especially in those varieties which are caused by transudation and by position. The following resumé of all that we know respecting this curious subject is given in the author's words, as translated in the work before us.

1. "There are some cases in which the ordinary signs of sanguineous congestion no sooner make their appearance, than the blood which flows to the organ affected escapes from its vessels, and hæmorrhage ensues. The signs of congestion cease as the blood flows, and the general health is in no way deranged, provided the hæmorrhage occurs in an organ of minor importance, or that it be the result of a natural process, such as the menstrual discharge. If, however, the organ be one whose functions cannot be deranged with impunity, the general health will suffer a proportionate derangement, as in the case of apoplexy or hæmoptysis. In the latter disease, however, the health may be re-established, provided the whole quantity of the blood effused be spit up; but if the blood be only partially thrown up, or if it all remain effused into the pulmonary parenchyma, the derangement of the general health will be permanent; as is exemplified in cases of extensive pulmonary apoplexy. When the bodies of persons dying during an attack of hæmorrhage are examined, the organs from which the blood was effused are sometimes found red and congested, whilst in other cases they are remarkably pale, and, with the exception of the blood effused, present no morbid alteration whatever. Such, for instance, is the appearance often presented in cases of hæmorrhage from the brain, bronchia, stomach, and intestines. This pale state of the bleeding tissues cannot be admitted as proof that no congestion had existed either before or during the hæmorrhage; all that it establishes is the simultaneousness of the afflux of blood to the tissue, and of its escape from its vessels.

2. Blood may for some time continue to accumulate in the vessels of a tissue, without making its escape; and in consequence of this accumulation, gives rise to various functional and organic derangements; but the hæmorrhage does not commence until a later period, when all the symptoms of inflammation have been fully developed. In all such cases, the quantity of blood effused is never so great as in the ordinary forms of hæmorrhage, as will readily appear by instituting a comparison between pneumonia and hæmoptysis, dysentery and melæna, &c.

3. When an organ has been irritated, the blood which flows thither in consequence of that irritation, may at first escape from its vessels in large quantities, and subsequently cease to be effused; and it is at this latter period when the hæmorrhage ceases, that the most formidable symptoms generally occur. In such cases the hyperæmia has only changed its form, but still persists; sometimes assuming an acute type, and advancing rapidly towards its termination, whether favourable or fatal; sometimes, also, retaining a latent chronic character, and laying the foundation of various alterations of nutrition in the organ where it exists. Thus hæmoptysis is sometimes transformed into pneumonia, hæmatemesis into gastritis, menorrhagia into metritis, &c.; thus also these hæmorrhages, after recurring frequently, and leaving some trace of hyperæmia after them at each visit, lay the foundation for the development of tubercles in the lungs, and of that degeneration, termed cancer, in the stomach and uterus.

4. We have just seen that hæmorrhage is in some cases the source from which various chronic alterations of nutrition derive their origin; it is in others evidently the effect of some of these alterations of nutrition; such for instance as induration, ulceration, accidental productions, &c.: around which, congestions, possessing a peculiar tendency to terminate by hæmorrhage, are frequently formed.

5. There are some active hyperæmias, which, whether existing simply, or combined with other organic derangements, have never been accompanied with the slightest extravasation of blood, either at their commencement, during their progress, or at their final termination.

6. Morbid anatomy does not afford any distinctive characters between the *post-mortem* appearances, presented by those cases which were attended with hæmorrhage, and those which were not.

We know not what the peculiar modification is, which the texture of an organ undergoes, so that in one case it allows the blood determined towards it to escape from its vessels; in another forms pus, or exhales only a thin serum; whilst in a third it becomes indurated, softened, or ulcerated: but there is one common link which unites these different alterations, and hence it is, that under the influence of apparently the same causes, we often see them produced indifferently, and not unfrequently replaced one by the other. Thus an atttack of coryza may be ushered in by epistaxis, which may be succeeded, first by a total cessation of secretion from the affected membrane, next by a serous exhalation, and subsequently by a puriform exhalation, which in its turn may be succeeded by a return of the epistaxis, with which the disease first commenced, and by which it terminates. But in all this series of phenomena we can perceive, throughout the whole course of the irritation, one constant lesion, namely, the hyperæmia; and a succession of morbid alterations in the organic action of the tissue affected, producing alternately, hæmorrhage, cessation of all secretion, exhalation of serum and of pus, and, lastly, a recurrence of the hæmorrhage. I might also adduce here the well-known fact, that in many cases of inflammation of serous membranes, which present no perceptible difference either in their nature, duration, or intensity, pure blood is sometimes found effused, sometimes a simple albuminous fluid, and sometimes perhaps false membranes, &c.

Thus from the observation of symptoms, as well as from the inspection of the part from which the blood flows, (which can be made in cases of cutaneous hæmorrhage) we arrive at this conclusion, that *active* hæmorrhage is uniformly preceded by the sanguineous congestion of the organ or tissue affected. But are we hence authorized to conclude that every species of hæmorrhage depends on increased vascular action? In my opinion, certainly not; for we can easily understand how certain hæmorrhages may depend exclusively on some modification of the organic disposition of the coats of the vessels, in consequence of which they may allow the blood to escape. Such a modification surely may exist without depending more on a sthenic, than an asthenic state of the vessels: indeed this fact appears to me abundantly proved by those cases of hæmorrhage which yield to the application of astringents, after bloodletting and other antiphlogistic measures have been repeatedly employed in vain." 89.

We have now brought our analysis of hyperæmia to a close, and trust that the article will not be unacceptable to those (and they form a large majority) of our readers, who have no opportunity of consulting the original. We hope, however, that this specimen of the work will induce many to purchase and peruse it, who would not otherwise do either.

